# Supplementary material for: Chromothripsis during telomere crisis is independent of NHEJ, and consistent with a replicative origin
Source: Genome Res. 2019 May;29(5):737–49. doi: 10.1101/gr.240705.118 (PMC6499312; doi:10.1101/gr.240705.118)
Supplement: Supplemental Material [file supp_gr.240705.118_Supplemental_file_1.zip › contigs/annotated_contigs/DB98/contig.2.DB98_length_443_mean_cov_4.40180586907.docx]

**DB98_length_443_mean_cov_4.40180586907**

```
AAAATAAGTTCATAAGGCAATAAGTGTGAATGAATCAAAACCCGTATAAACAATTAAGGACTCAGAATGGAGTAAAAAGGAAATTCACC
 >chr3:18222649-18222764 - E=1e-54
TCCATTAACAACTAAGTGACACTTAG|TTGTCT|ACAAAAGTTTGATTTTGTTGTTGTTGTTGTTGTTGTTTTAACTGATATACAGTAT
                                 >chr3:18221418-18221740 - E=2e-179
ACAGTGAACAAGAAAGTACTTACTGTTCCTTTTTGCTTGAATGCCCTGATTAAAAACCCTCCCTCCCTATGTCACTCTGATGGTAACAC

TTTTGCAGTGGTGTTTTTGAGGAAAGAGAGAGGTGAAATCAAAATTGTTTTATACGAAATGAAGGAAATCAATTTTGACACGACACGAA

TCTAAATGAAACATCTTTCTTTAGAAGTAGGTAAGATGTGTGAAATGACAGCAGAACTGGAGGTGGAAGAAACTGCTCTAAAAGAACTG
```
